# Editorial: Genomic Approaches for Improvement of Understudied Grasses

**DOI:** 10.3389/fpls.2017.00976

**Published:** 2017-06-09

**Authors:** Keenan Amundsen, Gautam Sarath, Teresa Donze-Reiner

**Affiliations:** ^1^Department of Agronomy and Horticulture, University of Nebraska-LincolnLincoln, NE, United States; ^2^Grain, Forage and Bioenergy Research Unit, United States Department of Agriculture—Agricultural Research ServiceLincoln, NE, United States; ^3^Department of Biology, West Chester UniversityWest Chester, PA, United States

**Keywords:** biomass yield, genotypic diversity, grasses, stress tolerance, RNA-seq, proteomics, genomics, differential gene expression

Grasses are diverse, spanning native prairies to high-yielding grain cropping systems. They are valued for their beauty and useful for soil stabilization, pollution mitigation, biofuel production, nutritional value, and forage quality; grasses encompass the most important grain crops in the world. There are thousands of distinct grass species and many have promiscuous hybridization patterns, blurring species boundaries. Resources for advancing the science and knowledgebase of individual grass species or their unique characteristics varies, often proportional to their perceived value to society. For many grasses, limited genetic information hinders research progress. Presented in this research topic is a brief snapshot of creative efforts to apply modern genomics research methodologies to the study of several minor grass species.

Native or naturalized grass species offer unique adaptation advantages and often have better heat, drought, and salinity tolerance than recently introduced species. Genotypes with unique combinations of traits frequently arise and many offer unique and robust sources of stress resistance or desirable production characteristics, but identification of those plants is challenging. Al-Dakheel and Hussain present a novel method for field-evaluating and identifying salinity tolerance in buffelgrass (*Cencrhus ciliaris* L.). Using a field-pot system over three years, 12 salt tolerant, high dry biomass yielding buffelgrass accessions were identified. Salinity tolerance is important in modern agriculture and particularly for many alternative grasses as they are often managed on marginal lands. Yousfi et al. specifically assayed the role of WRKY genes in salinity tolerance in durum wheat (*Triticum turgidum* L.). Five WRKY-containing bacterial artificial chromosomes (BACs) were identified, sequenced, and annotated. Differential response in WRKY genes in salt sensitive vs. salt tolerant germplasm was observed suggesting their role in salt tolerance. Another interesting finding, common to many grasses was the observation that 74.6% of the sequenced BACs contained transposable elements, often found in high copy numbers in grasses and further complicating genetic studies. Yue et al. used a global transcriptome approach to identify transcripts differentially expressed between a waxy, drought sensitive cultivar of broomcorn millet (*Panicum miliaceum* L.) and one that was non-waxy and salt and drought tolerant. Yue et al. reported the first assembly of broomcorn millet and identified 292 differentially expressed transcripts between the studied cultivars. These transcripts may be important in the morphological differences associated with waxiness, and drought and salinity tolerance. In addition to salinity tolerance, drought tolerance is essential for grasses grown on marginal lands. Hybrids can form between Italian ryegrass (*Lolium multiflorum* Lam.) and tall fescue (*Festuca arundinacea* Schreb.) and are exploited to combine desirable traits from each species. Perlikowski et al. examined two hybrid forms differing in their photosynthetic capacity during drought stress and their ability for membrane regeneration following removal of the stress, offering insights into the role of metabolic alterations on drought tolerance and membrane recovery.

Amaradasa and Amundsen studied the interaction between a fungal pathogen, *Curvularia inaequalis*, and resistant and susceptible American buffalograss [*Buchloë dactyloides* (Nutt.) Engelm. *syn. Bouteloua dactyloides* (Nutt.) Columbus]. Their analysis led to the development of RNA-based markers that have potential to screen and identify sources of host resistance in the absence of an *in vivo* assay. These markers have the added advantage of being gene-based, avoiding some of the challenges associated with genetic studies in complex polyploid genomes. Genetic marker development and testing is an important early step in working with grass species lacking genomic resources. As a part of the Yue et al. study, they identified 35,216 simple sequence repeat sequences in broomcorn millet that could be developed into molecular markers. Graves et al. used single nucleotide polymorphic markers (SNPs) to develop KASP assays. The KASP assays were able to discriminate hybridization and self-fertilization events in populations of prairie cordgrass (*Spartina pectinata* Link), which is challenging in a species with diverse and complex inheritance patterns. The ability to exploit important traits from breeding populations is critically important in order to maximize their value. Grinberg et al. used perennial ryegrass (*Lolium perenne* L.) breeding populations as a model to compare different predictive models in a genomic prediction framework with a goal to ultimately improve several biomass, yield, and nutritional value traits. Miao et al. examined the role of histones, another important conserved protein family essential for genome stability in switchgrass (*Panicum virgatum* L.) by exploiting genome specific markers and transforming tobacco to confirm their functional role. Miao et al. confirmed that the histone genes being investigated could trigger cell death and their nuclear localization was critical for their function.

Biomass is critically important for grasses destined for use by the biofuel industry. Lignin, cellulose, and pectin are cell wall constituents that influence how grasses can be used for biofuel production. Rai et al. conducted a genome-wide analysis to identify genes associated with cell wall composition of sorghum [*Sorghum bicolor* (L.) Moench]. By physically mapping those genes to the sorghum genome, researchers can use that information to alter cell wall composition through traditional plant breeding methods. Muthamilarasan et al. also examined gene expression profiles in cell wall associated genes in foxtail millet (*Setaira italica* L.) and identified genes differentially expressed in response to abiotic stress and exogenous hormone applications. Muthamilarasan et al. further describe the importance of synteny among grasses and conservation among cell wall genes. The study of Paudel et al. also highlights conservation among the grasses by studying expressed proteins during senescence in switchgrass and prairie cordgrass. Early senescence reduces biomass and therefore is an important process to understand in perennial grasses. By comparing early senescence genotypes with late senescence genotypes in both species, proteins intimately involved in senescence were found and could be exploited in future studies and breeding programs to develop germplasm with delayed senescence.

Here we highlight 11 understudied grass species, relative to the major cereal grasses, which is only a small fraction of the thousands of known species. As land resources become scarce and demand for highly productive arable land increases, identification of understudied grasses and their desirable traits can fast-forward their suitability for use in marginal lands. The papers presented in this research topic demonstrates novel approaches or new applications for proven methods to improve our understanding of perennial grasses and their function and illustrates important steps toward use of these understudied species in modern agriculture.

## Author contributions

All authors listed, have made substantial, direct and intellectual contribution to the work, and approved it for publication.

### Conflict of interest statement

The authors declare that the research was conducted in the absence of any commercial or financial relationships that could be construed as a potential conflict of interest.

